# Postural crisis in patients undergoing canalith repositioning procedures for posterior canal benign paroxysmal positional vertigo: A systematic review and meta-analysis

**DOI:** 10.1097/MD.0000000000040307

**Published:** 2025-01-17

**Authors:** Yee-Hyuk Kim, Hee-Jun Park, Jae-Ho Yoo

**Affiliations:** a Department of Otorhinolaryngology-Head & Neck Surgery, Daegu Catholic University School of Medicine, Daegu, Korea; b Department of Otorhinolaryngology-Head & Neck Surgery, Daegu Catholic University Medical Center, Daegu, Korea.

**Keywords:** benign paroxysmal positional vertigo, crisis, maneuver, meta-analysis, posture

## Abstract

**Background:**

The Epley or Semont maneuver is performed for posterior canal benign paroxysmal positional vertigo (PC-BPPV). The postural crisis indicates the phenomenon that the patient experiences severe dizziness, is unable to maintain the sitting posture, and suddenly falls backward or sideways on the examination table when returning to the sitting position, which is the final step of the canalith repositioning procedure (CRP). The postural crisis increases the risk of falls during CRP. This meta-analysis aimed to determine the incidence of postural crisis among patients who underwent CRP for PC-BPPV.

**Methods:**

Literature searches were conducted on PubMed, Embase, Cochrane Library, Web of Science, and Google Scholar. The random-effects model was utilized based on the results of the heterogeneity using the Tau^2^ test and I^2^ statistic.

**Results:**

We included 5 nonrandomized studies that reported the total number of cases of CRP for PC-BPPV and postural crisis. In each study, postural crisis occurred in 4.0% to 14.9% of the subjects. Of a total of 1177 cases, the incidence of postural crisis in all CRP cases was 10% (95% confidence interval = 6%–15%).

**Conclusion:**

BPPV is the most common among peripheral vestibular diseases, usually occurring in the posterior semicircular canal. Therefore, 10% of cases with CRP for PC-BPPV is significant. When performing CRP for PC-BPPV, considering that the postural crisis related to increasing the risk of falls may occur, preparations for the phenomenon should be made in advance.

## 1. Introduction

Benign paroxysmal positional vertigo (BPPV) occurs when otoconia, which are normally present in the utricle, enter the semicircular canal. When the head position changes, the otoconia within the semicircular canal moves to a dependent position under the influence of gravity. This produces endolymph currents within the semicircular canal and causes deflection of the cupula. These produce nystagmus and the patients experience as rotational vertigo. Theoretically, BPPV can occur in any of the 3 semicircular canals. Furthermore, both canalolithiasis and cupulolithiasis can occur in each semicircular canal. However, according to the 2015 Consensus document of the Committee for the Classification of Vestibular Disorders of the Bárány Society,^[1]^ the diagnostic criteria for canalolithiasis of the posterior canal (PC) and that of canalolithiasis and cupulolithiasis of the horizontal canal have been established. Canalolithiasis of the anterior canal, cupulolithiasis of the PC, and lithiasis of multiple canals are classified as emerging and controversial syndromes. BPPV has the highest incidence among peripheral vestibular diseases.^[1,2]^ The most common form of BPPV is canalolithiasis of the posterior semicircular canal (PSC).^[1,3,4]^

Different canalith repositioning procedures (CRPs) are performed for each type of BPPV. CRP is the most effective treatment for BPPV because it allows the otoconia within the semicircular canal to return to the utricle via a change in head and body posture. The patients in this study had BPPV due to canalolithiasis of the PSC, and the Epley or Semont maneuver was used for its treatment. In both these procedures, the final patient position is the same, which is a return to the sitting position.^[5–9]^

During the last step of CRPs for posterior semicircular canal BPPV (PSC-BPPV), down-beating nystagmus may occur, leading to severe dizziness, inability to maintain the sitting posture, and eventually sudden fall backward or sideways on the examination table. This phenomenon has been considered panic, Tumarkin-like phenomenon, trunk retropulsion, postural control loss, or anterior canal crisis^[3,10–13]^; in this study, it is referred to as a postural crisis. A down-beating nystagmus occurs just before the postural crisis develops. During the last step of the Epley and Semont maneuver, if the otoconia fail to return to the utricle and remain in the PSC when returning to the sitting position, they move to the ampulla and cause down-beating nystagmus. A postural crisis does not develop in this situation, and this sign indicates treatment failure. Although the pathogenesis of postural crisis has not been clearly understood, it is presumed to occur when the otoconia of the PSC enters the utricle. Therefore, a postural crisis indicates a successful treatment outcome.^[3,10,12,14,15]^

When a postural crisis develops, patients may be alarmed. Furthermore, physicians are bound to be embarrassed because postural crisis is an unpredictable dangerous event. Moreover, postural crisis increases the risk of patients falling from the examination table during CRPs for PSC-BPPV. If a fall occurs during CRP, the patient will suffer physical damage. Despite this, there are few studies on this phenomenon. Ideally, before CRP, the patient must be informed that brief but very strong dizziness may occur during CRP, and physicians should also be conscious of this fact during CRP.

This study aimed to determine the incidence of postural crisis during CRPs in patients with PSC-BPPV. Thus, we performed a systematic review and meta-analysis.

## 2. Methods

### 2.1. Search strategy

Systematic literature review and meta-analysis were conducted according to the Preferred Reporting Items for Systemic Review and Meta-analyses guidelines.^[16]^ PubMed, Embase, Cochrane Library, Web of Science, and Google Scholar were searched from the date of inception to September 2, 2023. The search terms are listed in Data S1, Supplemental Digital Content, http://links.lww.com/MD/O269, which shows the search strategy and result in each database), and the language was restricted to English.

### 2.2. Inclusion and exclusion criteria

First, we searched for studies on CRP in patients diagnosed with PSC-BPPV. Then, we included only those that reported severe dizziness and the inability of the patients to remain sitting posture, resulting in sudden falling backward or sideways during the final stage of CRP. Therefore, the studies that described the number of patients diagnosed with PSC-BPPV and the number of patients who developed a postural crisis during the CRP among those patients, not limited to specific study designs, were included. We excluded studies that reported on the following: patients with PSC-BPPV though experiencing only vertigo without nystagmus induced by the Dix-Hallpike or side-lying test (i.e., subjective BPPV); patients experiencing severe dizziness and showing down-beating nystagmus when returning to the sitting position in CRP but did not report any failure to maintain the sitting posture nor falling backward; and the total number of patients who collapsed without loss of consciousness was not reported.

### 2.3. Data extraction and quality assessment

The primary outcome of this study was to determine the incidence of postural crisis from the total number of CRPs (Epley or Semont maneuvers) performed in BPPV of the PSC. 3 authors independently reviewed the included studies. The following data were extracted: the first author’s name, publication year, study location, study design, study subjects’ diagnosis and treatment method, the total number of cases that received CRP for PSC-BPPV, and the total number of cases in which a postural crisis occurred. In cases where there were inconsistent data, the reviewers resolved them through discussion. The Risk of Bias Assessment Tool for Nonrandomized Studies (RoBANS) was used for the quality assessment of nonrandomized studies.^[17]^

### 2.4. Statistical analyses

R-4.2.2 for Windows with R package “metafor” and “meta” was used for statistical analysis. The incidence of postural crisis with severe dizziness when patients with PSC-BPPV returned to the sitting position was determined: the number of cases with postural crisis/the total number of cases with CRP. The incidence was calculated with 95% confidence intervals. Heterogeneity between studies was evaluated using I^2^ statistics and tau-squared estimator.^[18,19]^ The tau-squared estimator estimates the variance of distribution of the true effect sizes. The I^2^ statistic attributes the total percentage of variance among studies to heterogeneity rather than sampling chance. The I^2^ inconsistency index, whose values are classified as low at 25%, moderate at 50%, or high at 75%, was adopted.^[20]^

### 2.5. Ethical consideration

Owing to the nature of the study, that is, published data were used and did not include individualized patient data, an Institutional Review Board screening was optional.

## 3. Results

### 3.1. Systematic literature review

**Figure 1.**
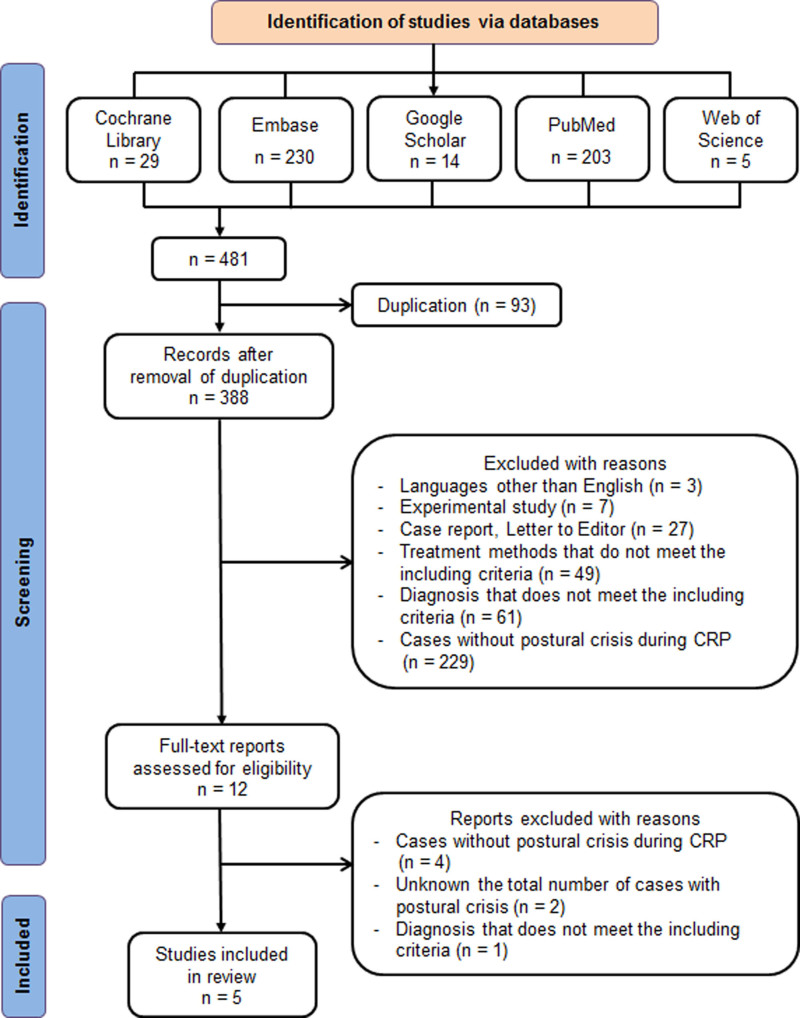
Study selection process according to the preferred reporting items for systematic reviews and meta-analyses flow diagram.

A total of 482 potentially relevant studies were initially identified. The selected papers were 1 prospective and 4 retrospective observational studies. Only 5 were finally included in the analysis.^[3,10–13]^ The selection process is presented in Figure 1.

### 3.2. Characteristics of the studies and quality assessment

This research includes only cross-sectional studies. Table 1 presents the details of the 5 included studies, including the total number of cases, the total number and frequency of events in which postural crisis occurred, and suggestions from each study author to prevent or prepare for the postural crisis. The RoBANS, which consisted of 6 domains, was used to assess the risk of bias in the included studies (Table 2). Each domain of the RoBANS was evaluated according to the criteria for judgment in the Appendix section.^[17]^

**Table 1:** Summarized data of the included studies.

Year/Author	Country	Total cases	Event	Event/Total (%)	Description of the event	Author’s recommendation
2005 Uneri	Turkey	436	58	13.30	Falling sensation, panic, DBN, and unable to sit upright	Patients should be held tightly as they are moved to the sitting position
2018 Maranhao	Brazil	221	33	14.93	Falling sensation, sudden backward movement without loss of consciousness, and Tumarkin-like phenomenon	Hold on to the patient for at least 1 minute after repositioning
2020 Power	Australia	248	10	4.03	Brief intense vertigo (often falling sensation), DBN, and trunk retropulsion	Be vigilant and support the patient’s trunk during the sitting up phase of the EM in the event
2022 Kim	South Korea	94	14	14.89	Severe dizziness (feeling of falling down), DBN, and postural control loss	Firmly secure the patient from immediately after the EM to the most prolonged latency period of the DBN
2023 Shigeno	Japan	178	14	7.87	Transient intense vertigo, DBN, retropulsion, and anterior canal crisis	Patient’s head should not be rotated beyond 135° to the healthy ear and the top of the head would not be gone down during EM

DBN = down-beating nystagmus, EM = Epley maneuver.

**Table 2:** Risk of bias assessment of nonrandomized studies using the RoBANS.

	Selection of participants	Confounding variables	Measurement of exposure	Blinding of outcome assessments	Incomplete outcome data	Selective outcome reporting
2005 Uneri	High	High	Low	Low	High	Low
2018 Maranhao	High	High	Low	Low	Low	Low
2020 Power	Unclear	High	Low	Low	Low	Low
2022 Kim	High	High	Low	Low	Low	Low
2023 Shigeno	Unclear	High	Low	Low	Low	Low

Each domain was classified as Unclear Risk, Low Risk, or High Risk.

RoBANS: Risk of Bias Assessment Tool for Nonrandomized Studies.

#### 3.2.1. Selection of participants

In RoBANS, the risk of bias is defined as low risk in “Selection of participants” if the study participants were consecutively recruited and the data were prospectively collected. Of the 5 included studies, 2 were evaluated as unclear: Power,^[11]^ a prospective study that did not describe whether the data were consecutively collected, and Shigeno,^[13]^ which did not clearly describe its recruitment method and data collection. The remaining 3 studies were evaluated as high risk because they retrospectively collected data from study subjects.

#### 3.2.2. Confounding variables

The domain “Confounding variables” was evaluated as high risk, since all 5 studies simply presented the total number of cases in which CRP was performed and the total number of cases in which postural crises occurred among them but did not use stratification or statistical adjustment.

#### 3.2.3. Measurement of exposure

The domain “Measurement of exposure” was evaluated as low risk because self-reported methods were not used in data collection, and data were collected from medical records without room for interviewer bias or recall bias.

#### 3.2.4. Blinding of outcome assessments

The domain “Blinding of outcome assessments” was assessed as low risk because postural crisis is an objective phenomenon that occurs regardless of the patient’s will, so blinding cannot affect the frequency of postural crisis.

#### 3.2.5. Incomplete outcome data

Considering the study period, Uneri^[10]^ performed their study before the BPPV of the PSC was classified as canalolithiasis or cupulolithiasis. Therefore, the domain “Incomplete outcome data” was evaluated as high risk due to the possibility that the study included not only patients with canalolithiasis of the PSC-BPPV but also cupulolithiasis when calculating the incidence of postural crisis. The remaining 4 studies included only canalolithiasis and excluded cupulolithiasis among PSC-BPPV, and thus these were evaluated as low risk.

#### 3.2.6. Selective outcome reporting

The domain “Selective outcome reporting” was considered low risk because the primary outcomes were planned and analyzed according to the research protocol.

### 3.3. Heterogeneity

**Figure 2.**
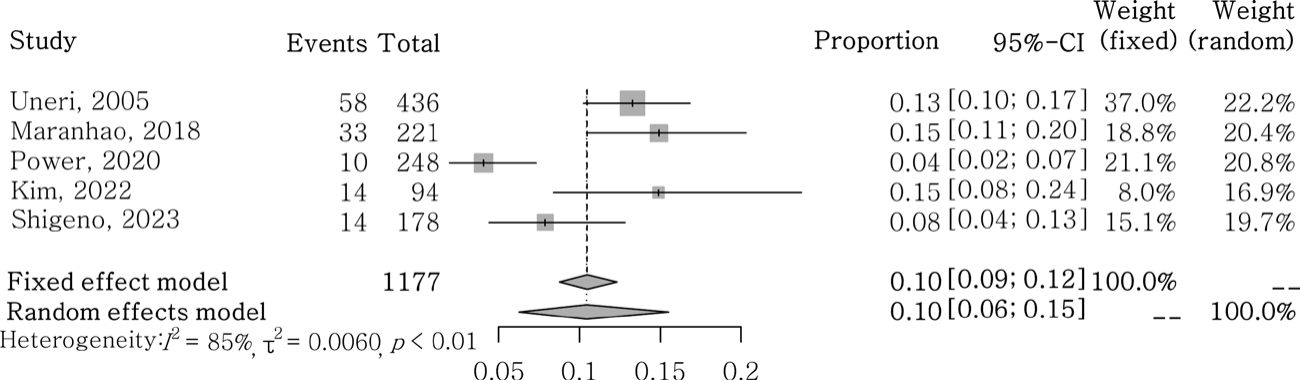
Forest plot showing the incidence of postural crisis among the cases involving the canalith repositioning procedure for benign paroxysmal positional vertigo of posterior semicircular canal.

**Figure 3.**
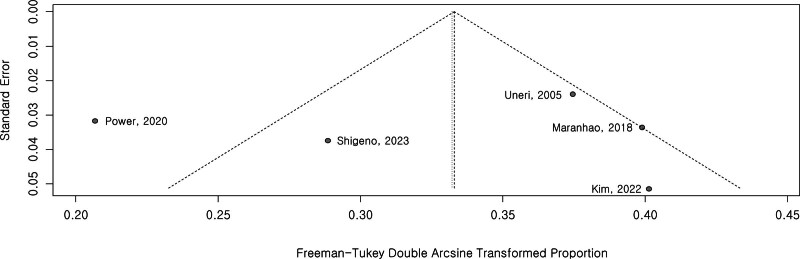
Funnel plot showing an unbiased incidence and diverse distribution of postural crisis and number of study subjects in 5 studies.

**Figure 4.**
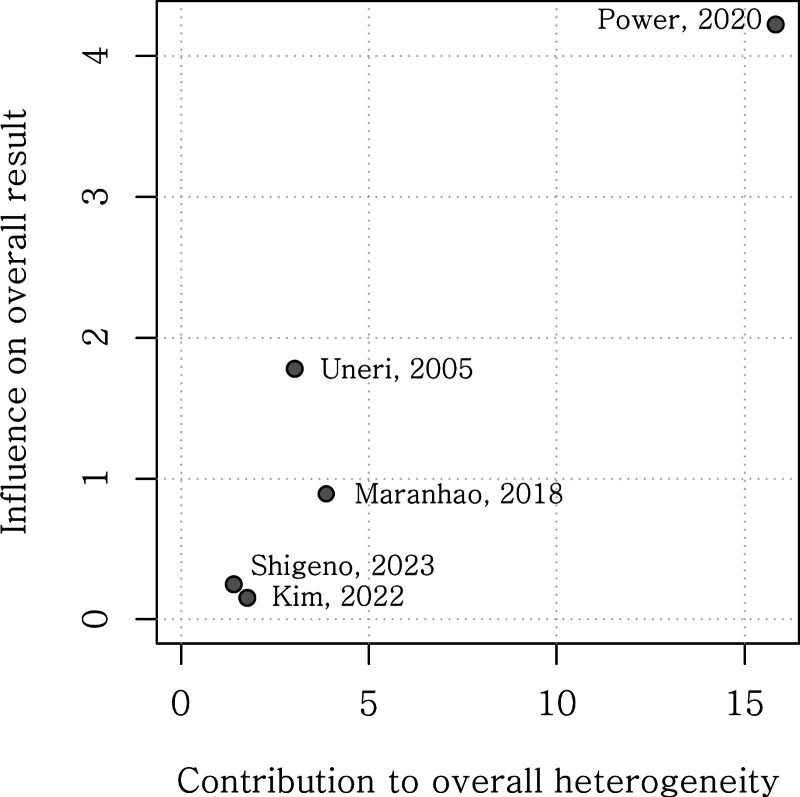
Baujat plot showing the influence on the overall result and contribution to the overall heterogeneity of studies included in the meta-analysis.

Data from 5 studies with 1177 participants were pooled. Heterogeneity among the studies selected was significant (*P* < .01; tau^2^ = 0.0060, I^2^ = 85%). Therefore, analyses were performed using the random effect model (Fig. 2). The funnel plot showed that the sample sizes and event rates of the 5 included studies were not biased and were evenly distributed (Fig. 3). Additionally, a Baujat plot illustrated the influence on the overall result and contribution of each study to the overall heterogeneity in the meta-analysis (Fig. 4). Excluding Power,^[11]^ 4 studies have relatively low heterogeneity.

### 3.4. Outcomes

Using the results of 5 studies, we analyzed the incidence of postural crisis in patients who underwent CRPs for PSC-BPPV. Overall, the total number of study subjects was 1177. When analyzed using a random-effects model based on the results of heterogeneity, the incidence of postural crisis in total CRP cases was 0.10 (95% confidence interval: 0.06–0.15) (Fig. 2).

## 4. Discussion

Of the 5 papers included in this meta-analysis, 4 articles were published from 2018 to 2023, and all subjects included in each study were diagnosed with canalolithiasis of the PC-BPPV.^[3,11–13]^ However, a paper published in 2005 was a retrospective analysis of patients from 1996 to 2001 and stated that the study subjects were diagnosed with PC-BPPV.^[10]^ This is thought to be because at the time the study was conducted, the diagnostic criteria and methods for distinguishing between canalolithiasis and cupulolithiasis in BPPV of the PSC were not clearly presented.^[1]^ The description of the 5 papers included in the present study about the postural crisis was similar in many aspects: patients reported said that they felt a brief but extreme dizziness; the dizziness that they felt was a falling sensation rather than rotating nature vertigo; the degree of dizziness was very severe but without loss of consciousness and often accompanied by strong down-beating nystagmus; and the patients were unable to maintain a sitting posture and suddenly fell backward or sideways. The articles have referred to this phenomenon as panic, being unable to sit upright,^[10]^ sudden backward movement, loss of seated postural stability, Tumarkin-like phenomenon,^[3]^ trunk retropulsion, otolithic crisis or catastrophe,^[11]^ postural control loss,^[12]^ and retropulsion or unpredictable anterior canal crisis.^[13]^ Power^[11]^ investigated the incidence of loss of body balance in returning to the sitting position of CRP and falling backward or sideways on the examination table and reported a Tumarkin-like phenomenon in 45 cases of CRP for 248 PSC-BPPV patients, but trunk retropulsion occurred in only 10 of these patients.^[11]^ In this meta-analysis, the number of patients who experienced trunk retropulsion in the study of Power^[11]^ was included. Postural crisis might have occurred when the otoconial mass in the PSC passed through the common crus of the semicircular canal (effect on the anterior semicircular canal) or entered the utricle (effect on the macula of the utricle).^[3,10,12,17,21,22]^ During postural crisis, patients do not complain of rotational vertigo but rather feel a falling sensation and down-beating nystagmus, making such predictions quite reasonable. In cases of postural crisis during CRP, weight, size, and moving velocity of the otoconial mass would be different from those in cases where it does not occur.^[3,10,12,17,21,22]^

When down-beating nystagmus occurs during the final phase of the CRPs for PSC-BPPV, if the otoconia do not return to the utricle and remain in the semicircular canal, they move to the ampulla, causing nystagmus and dizziness. In such patients, the severe postural imbalance corresponding to a postural crisis does not appear, indicates treatment failure. However, if a postural crisis occurs when down-beating nystagmus develops in the same position, the intensity of the nystagmus is much stronger and the sense of imbalance is more severe. This makes it impossible for the patients to maintain a sitting position on the examination table. At this time, the patients are greatly startled, and they scream due to the abrupt and severe sense of imbalance. The patients equated this sensation to that of being pulled into the ground or falling down. These descriptions indicate that the patients experience a strong feeling of moving vertically downward rather than rotational dizziness. Thus, postural crisis may be a utricle-related phenomenon, and the treatment may yield a successful outcome if the patients experience postural crisis. A down-beating nystagmus occurs just before the postural crisis develops. Therefore, doctors should not immediately take off the patient’s goggles nor leave the patient from behind immediately after the patient returns to a sitting position in the Epley or Semont maneuver. In order to safely support the patient and prevent a fall from the examination table due to a severe imbalance, physicians should remember that a postural crisis may occur immediately after the CRPs for PSC-BPPV, and it may be preceded by down-beating nystagmus.

To prevent or deal with postural crisis, the authors of the 5 included studies proposed the following. Shigeno^[13]^ suggested the modification of the Epley maneuver posture as a preventive method: In the third head position of the Epley maneuver, the patient’s head is held stably with the examiner’s palm, the patient’s head is not turned more than 135° toward the healthy ear, and the top of the patient’s head is not allowed to go down.^[13]^ Shigeno^[13]^ reported that they were able to reduce the frequency of mixed downbeat and torsional nystagmus and retropulsion in the fourth head position.^[13]^ The other 4 studies suggested countermeasures to prevent patients from falling down. When returning the patient to the sitting position during CRP, the patient must be held tightly for a certain period of time.^[3,10–12]^ Practitioners who perform CRP must be mindful of the risk of falling off the examination table; the minimum amount of time that a patient should be held on has also been suggested. According to Kim,^[12]^ down-beating nystagmus occurs before the postural crisis. The most prolonged latency of down-beating nystagmus was 21 seconds.^[12]^ To determine whether downbeat nystagmus occurs in the final phase of CRP, the patient should be tightly held and observed beyond the most prolonged latency period of down-beating nystagmus. In addition, when down-beating nystagmus occurs, there is a high possibility that the postural crisis will follow.^[12]^ According to Maranhao,^[3]^ in most cases, this situation occurs within 1 minute after returning to the sitting position, so the time to tightly hold the patient should be at least 1 minute.^[3]^ The postural crisis is presumed to be related to the otoconial mass passing through the common crus of the semicircular canal and entering the utricle. Thus, the occurrence of postural crisis may indicate a successful treatment outcome.^[3,10,12,17,21,22]^ Awareness of the possibility of postural crisis may help physicians to prepare for it and thereby reduce the patient’s fear of sudden dizziness and prevent falls. Therefore, this ensures physical safety and psychological stability of the patients, and successful treatment results can be expected.

## 5. Conclusion

When returning to the sitting position in the CRP, the postural crisis may occur in 4% to 14.9% of cases in individual studies, showing relatively diverse results. On meta-analysis, the postural crisis occurred in 10% of CRP for PSC-BPPV. Since the PSC-BPPV occurs most frequently among peripheral vestibular diseases, an incidence of 10% is significantly high. Postural crisis indicates an increased risk of falling off the examination table and physical damage. Therefore, clinicians must be aware that this phenomenon may occur when performing CRP and prepare in advance.

## Author contributions

**Conceptualization:** Yee-Hyuk Kim.

**Data curation:** Yee-Hyuk Kim, Hee-Jun Park, Jae-Ho Yoo.

**Formal analysis:** Yee-Hyuk Kim.

**Methodology:** Yee-Hyuk Kim, Hee-Jun Park, Jae-Ho Yoo.

**Writing – original draft:** Yee-Hyuk Kim.

**Writing – review & editing:** Yee-Hyuk Kim, Hee-Jun Park, Jae-Ho Yoo.

## Supplementary Material


