# Supplementary material for: Postural crisis in patients undergoing canalith repositioning procedures for posterior canal benign paroxysmal positional vertigo: A systematic review and meta-analysis
Source: Medicine (Baltimore). 2025 Jan 17;104(3):e40307. doi: 10.1097/MD.0000000000040307 (PMC11749583; doi:10.1097/MD.0000000000040307)
Supplement: Supplementary file 1 [file medi-104-e40307-s001.docx]

Supplemental content for search strategy and result in each database

1. Search strategy in PubMed

| Search query | | Items found |
| --- | --- | --- |
| #1 | benign paroxysmal positional vertigo or BPPV | 2,913 |
| #2 | posterior semicircular canal or posterior canal | 11,261 |
| #3 | canalith repositioning maneuver or canalith repositioning procedure | 413 |
| #4 | Epley maneuver or Semont maneuver | 425 |
| #5 | downbeat nystagmus or down-beating nystagmus | 654 |
| #6 | falling sensation or Tumarkin or retropulsion | 8,571 |
| #7 | anterior semicircular canal or utricle or otolith | 8,183 |
| #8 | #1 and #2 | 842 |
| #9 | #3 or #4 | 731 |
| #10 | #6 or #7 | 16,701 |
| #11 | #5 and #8 and #10 | 33 |
| #12 | #9 and #10 | 180 |
| #13 | #11 or #12 | 203 |

2. Search strategy in Embase

| Search query | Items found |
| --- | --- |
| ('benign paroxysmal positional vertigo'/exp OR 'benign paroxysmal positional vertigo' OR (benign AND paroxysmal AND positional AND ('vertigo'/exp OR vertigo)) OR bppv) AND ('posterior semicircular canal'/exp OR 'posterior semicircular canal' OR (posterior AND semicircular AND canal) OR 'posterior canal' OR (posterior AND canal)) AND ('canalith repositioning maneuver'/exp OR 'canalith repositioning maneuver' OR (('canalith'/exp OR canalith) AND ('repositioning'/exp OR repositioning) AND maneuver) OR 'canalith repositioning procedure'/exp OR 'canalith repositioning procedure' OR (('canalith'/exp OR canalith) AND ('repositioning'/exp OR repositioning) AND ('procedure'/exp OR procedure)) OR 'epley maneuver'/exp OR 'epley maneuver' OR (epley AND maneuver) OR 'semont maneuver'/exp OR 'semont maneuver' OR (semont AND maneuver)) AND ('downbeat nystagmus'/exp OR 'downbeat nystagmus' OR (downbeat AND ('nystagmus'/exp OR nystagmus)) OR 'down-beating nystagmus' OR ('down beating' AND ('nystagmus'/exp OR nystagmus)) OR 'falling sensation' OR (('falling'/exp OR falling) AND ('sensation'/exp OR sensation)) OR 'panic'/exp OR panic OR tumarkin OR 'retropulsion'/exp OR retropulsion OR 'anterior semicircular canal'/exp OR 'anterior semicircular canal' OR (anterior AND semicircular AND canal) OR 'utricle'/exp OR utricle OR 'otolith'/exp OR otolith) | 230 |

3. Search strategy in Google scholar, Web of Science, and Cochrane library

| Boolean operators | Search engine | Items found |
| --- | --- | --- |
| (((downbeat nystagmus or down-beating nystagmus) AND ((benign paroxysmal positional vertigo or BPPV) AND (posterior semicircular canal or posterior canal))) AND ((falling sensation or Tumarkin or retropulsion) OR (anterior semicircular canal or utricle or otolith))) OR (((canalith repositioning maneuver or canalith repositioning procedure) OR (Epley maneuver or Semont maneuver)) AND ((falling sensation or Tumarkin or retropulsion) OR (anterior semicircular canal or utricle or otolith))) | Cochrane library | 29 |
|  | Google scholar | 14 |
|  | Web of Science | 5 |
